# An Experimental Study on the Mechanical Properties and ANN-Based Prediction of a Tensile Constitutive Model of ECCs

**DOI:** 10.3390/polym17233183

**Published:** 2025-11-29

**Authors:** Qi Zhao, Zhangfeng Yang, Xiaofeng Zhang, Zhenmeng Xia, Kai Xiong, Jin Yan

**Affiliations:** 1 College of Ocean Engineering and Energy, Guangdong Ocean University, Zhanjiang 524088, China; 2 Guangdong Provincial Key Laboratory of Intelligent Equipment for South China Sea Marine Ranching, Guangdong Ocean University, Zhanjiang 524088, China; 3 Naval Architecture and Shipping College, Guangdong Ocean University, Zhanjiang 524088, China

**Keywords:** Engineered Cementitious Composites (ECCs), mechanical properties, Artificial Neural Network (ANN), microscopic analysis, tensile constitutive model

## Abstract

Traditional concrete materials have limitations in terms of load-bearing capacity and ductile failure. In contrast, Engineered Cementitious Composites (ECCs), with their superior strain-hardening behavior and multiple cracking characteristics, have attracted widespread attention in the field of high-performance materials. In this study, ECC specimens incorporating different types of fibers (polyethylene (PE) fibers, polyvinyl alcohol (PVA) fibers) at varying contents were tested to systematically analyze their influence on mechanical properties. Compressive, flexural, and uniaxial tensile strength tests were conducted to evaluate the mechanical performance of ECCs. In addition, scanning electron microscopy (SEM) was employed to examine the fracture surfaces of the fibers, providing deeper insights into the interfacial behavior and fracture morphology of the different fiber-reinforced systems. Fracture surface analysis reveals that the interfacial bonding characteristics between different fibers and the matrix significantly influence fracture behavior. Moreover, as the tensile performance of ECCs is influenced by the interaction of multiple factors, traditional constitutive models exhibit limitations in accurately predicting its complex nonlinear behavior. To address this limitation, an Artificial Neural Network (ANN) approach was adopted to develop a predictive model based on bilinear stress–strain relationships. The model was constructed using ten key input parameters, including matrix composition and fiber properties, and was able to accurately predict the first cracking strain, first cracking stress, ultimate strain, and ultimate stress of ECCs. Sensitivity analysis revealed that fiber tensile strength and fiber content were the most significant factors influencing the tensile behavior. The predicted tensile curves showed strong consistency with the experimental results, thereby confirming the reliability and applicability of the proposed ANN-based model.

## 1. Introduction

With the increasing performance demands placed on materials in modern engineering structures, the limitations of traditional concrete in ductility, crack control, and durability have become apparent, making it difficult to satisfy the demands of high-performance service environments [1,2]. Particularly in coastal regions, hydraulic structures are exposed to seawater over extended periods, making concrete materials prone to performance degradation. Common issues include crack propagation, interfacial damage, and strength deterioration, all of which significantly compromise the durability and safety of the structure. Therefore, effective strengthening and repair of concrete structures are essential. ECCs, as novel high-ductility cement-based materials, exhibit strain-hardening behavior and multiple cracking behavior, with ultimate tensile strains reaching 3–7% [3,4,5]. In recent years, ECCs have emerged as a research focus in the field of strengthening and repairing marine engineering structures. originally proposed by Li et al. [6]. ECCs leverages the synergistic interaction between fibers and the cementitious matrix to maintain high load-bearing capacity even after crack initiation, thereby enhancing overall toughness and resistance to failure. However, the mechanical properties of ECCs are influenced by a variety of factors; the fiber types, content, and surface modification are critical parameters [7,8]. In practical engineering applications, PE and PVA fibers have been widely used in the material design and performance optimization of ECCs due to their ability to improve crack resistance, ductility, and overall toughness. As a result, they have become the principal fiber types for performance enhancement in ECCs.

The type and content of fibers are critical factors influencing the mechanical properties of ECCs. As bridging elements across microcracks, fibers have a significant influence on the crack resistance, ductility, and strength of ECCs [9,10]. Existing studies have shown that PE fibers, owing to their high modulus, high strength, and excellent toughness, are effective in enhancing the tensile strength of ECCs [11]. However, due to their smooth surface, PE fibers primarily rely on frictional slip mechanisms for fiber–matrix interfacial bonding [12]. In contrast, the PVA fibers, particularly the RECS-15 product developed by Kuraray Co., Ltd. in Japan, exhibit a better interfacial bonding performance due to hydrophilic surface treatment [13]. By comparison, untreated PVA fibers exhibit relatively poorer performance in terms of interfacial bond strength, tensile strength, and elastic modulus [14,15,16]. Regarding fiber content, Wang et al. [11] observed that increasing the PE fiber content from 1% to 2% resulted in pronounced strain-hardening behavior and a well-defined pattern of multiple cracks in ECCs. However, a further increase to 3% led to a significant reduction in both tensile and compressive performance. Khan et al. [17], Ling et al. [18] and Kang et al. [19] reported that when the content of PVA fibers exceeded a certain threshold (e.g., 1.5%), performance deterioration was observed, primarily attributed to fiber agglomeration and inadequate dispersion. Ma et al. [20] further emphasized that excessive fiber content can negatively impact the overall mechanical properties of ECCs.

Recent studies have demonstrated that surface modification of fibers can markedly enhance the mechanical properties and durability of ECCs. Gurbuz et al. [21] utilized nano-graphite to treat PVA fibers, resulting in significant improvements in the tensile strength, toughness, and impact resistance of the composite. Al-Baghdadi et al. [22] showed that appropriate interfacial treatments effectively improve fiber–matrix bond strength. Sun et al. [23] applied systematic surface modification techniques to improve the interfacial adhesion between PVA fibers and the matrix, thereby achieving superior ductility and crack control. Similarly, Ding et al. [24] coated PVA fibers with various chemical agents and confirmed that optimized fiber–matrix interactions contribute to enhanced deformability of ECCs. Although various studies have investigated the effects of fiber surface treatments on the interfacial bonding and mechanical properties of ECCs, the characterization of interfacial behavior and the associated micromechanical mechanisms remain limited. Therefore, an in-depth investigation into the interfacial behavior and mechanical properties of both treated and untreated PVA fibers is essential for guiding the rational selection of fiber types and developing effective interfacial optimization strategies in ECCs.

Traditionally, the mechanical performance of ECCs has been studied primarily through extensive experimental testing to obtain stress–strain curves. However, such experiments are often time-consuming, labor-intensive, and costly, and it remains difficult to comprehensively explore all possible combinations of material parameters. In practical engineering applications, multiple trials are often required to determine the optimal mix design, which, to some extent, limits the widespread adoption and application of ECCs. Consequently, developing an efficient model to accurately predict the tensile performance of ECCs. Therefore, the establishment of a model that enables efficient prediction of the tensile performance of ECCs has become a major focus of current research [25,26].With the rapid advancement of artificial intelligence technologies, machine learning (ML) methods have increasingly demonstrated their advantages in the study of civil engineering materials [27]. Recent studies have shown that machine learning can effectively predict the mechanical properties of alternative cementitious materials such as geopolymers (for example, coal gangue-based geopolymers), and can be used to optimize mix proportions and reduce experimental costs, thereby providing important guidance for the modeling strategy adopted in this study [28]. Among the various machine learning methods, Artificial Neural Network (ANN), as a representative ML algorithm, can effectively learn complex input–output relationships from training data, enabling accurate prediction of previously untested material properties. Compared with traditional empirical formulas or linear regression models, ANN models not only deliver higher predictive accuracy but also excel in capturing the intricate nonlinear behaviors resulting from the coupling of multiple influencing factors. This capability significantly enhances their adaptability and effectiveness across a wide range of material systems [29,30].

In previous studies, in addition to data-driven modeling approaches, numerical simulation methods such as finite element analysis and damage–plasticity models have also been widely used to simulate the tensile behavior and damage evolution of ECCs. These models are capable of describing crack initiation, propagation, and evolution from a micromechanical perspective and exhibit strong applicability at the structural level [31,32]. However, such methods typically rely on complex material constitutive relationships and highly refined parameter calibration, which limits their engineering practicality in material design and performance assessment. By contrast, ANN models can efficiently predict key tensile performance indicators of ECCs on the basis of material parameters with clear physical meaning and can output engineering-oriented bilinear stress–strain curves, thereby providing a useful reference for ECCs performance design and structural member analysis. Nevertheless, existing ANN–ECCs studies still have notable limitations: for the input parameters, most rely on empirical selection or statistical correlations, making it difficult to capture the underlying physical mechanisms governing the evolution of material properties [33]; furthermore, the majority of studies focus on a single fiber system (mainly PVA) and lack validation of predictive performance across different fiber systems [34].

In summary, based on previous research findings, a systematic experimental program was designed to investigate the effects of different fiber types and varying fiber content on the mechanical properties of ECCs through compressive, flexural, and uniaxial tensile tests. In the compressive, flexural and uniaxial tensile tests, the bridging mechanisms and effects of the various fibers were revealed through macroscopic destruction form and changes in strength. Additionally, by observing the fracture surfaces of the fibers and the propagation of cracks, a thorough analysis was conducted on the influence of the fiber-matrix interface on the destruction form of the specimen. In addition, an ANN-based modeling framework was developed to predict the tensile behavior of ECCs. With respect to the input parameters, in light of the material composition and governing factors of ECCs’ tensile behavior, parameters with clear physical meaning were selected, including fiber mechanical properties, fiber diameter, length, content, and matrix mix-design–related constituents. Furthermore, ANN-based sensitivity analysis was performed to quantify the influence of different parameters on tensile performance, thereby enhancing the interpretability of the model. Moreover, literature data and experimental data were combined to construct a mixed dataset covering PVA, PE, and PP fiber systems, and a stratified error analysis was conducted for the main fiber systems with sufficient data (PVA and PE) to evaluate cross-system predictive performance. To further improve predictive accuracy, separate single-output ANN models were established for the four tensile parameters, accompanied by systematic hyperparameter optimization. These approaches enhance the generalization capability of the model for the main fiber systems and provide a methodological basis for extending ECCs tensile performance prediction to a wider range of fiber systems in future studies.

## 2. Materials and Methods

### 2.1. Materials

**Figure 1:**
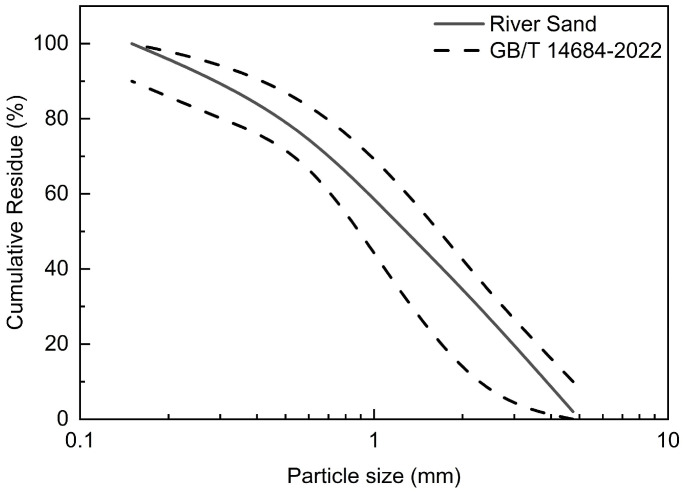
Gradation curve of river sand.

The Zhanjiang-produced Conch brand P.O. 42.5 ordinary Portland cement (OPC) (Zhanjiang, China) and fly ash from Shijiazhuang Shang’an Power Plant (Shijiazhuang, China) were used as the binder materials for preparing ECCs. The chemical composition of the binder materials is shown in Table 1. According to the ASTM C618-25a standard [35], the fly ash used in the experiment is classified as Class F. River sand with an average particle size of 150 μm was used as the fine aggregate, and the particle gradation curve of the river sand is shown in Figure 1, which complies with the grading requirements for building sand specified in GB/T 14684-2022 [36]. A polycarboxylate-based superplasticizer (HLX, standard type) manufactured in Shanxi Province was employed, with a water reduction rate of 27%.

**Figure 2:**
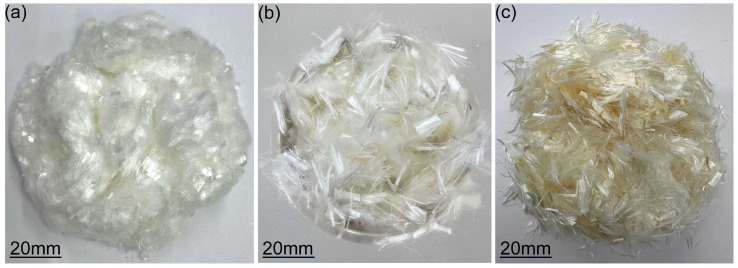
(**a**) PE fibers; (**b**) K-PVA fibers; (**c**) N-PVA fibers.

Figure 2 shows different types of fibers: PE fibers, the Kuraray PVA (K-PVA) fibers, and untreated PVA (N-PVA) fibers. The PE fibers were produced in Zibo, Shandong Province, China. The K-PVA fibers, coated with an oiling agent to improve dispersion and fiber–matrix interaction, were of the RECS-15 type produced by Kuraray Co., Ltd. (Japan), whereas the N-PVA fibers were provided by a manufacturer located in Tai’an, Shandong Province, China. The physical and mechanical properties of the fibers are listed in Table 2.

The mix proportions of ECCs are shown in Table 3. In the initial stage of the experiment, based on previous experience and preliminary test results, a water-to-binder ratio of 0.27, a sand-to-binder ratio of 0.36, and a fly ash-to-cement ratio of 1.2:1 were adopted.

### 2.2. Specimen Preparation, Curing, and Testing

**Figure 3:**
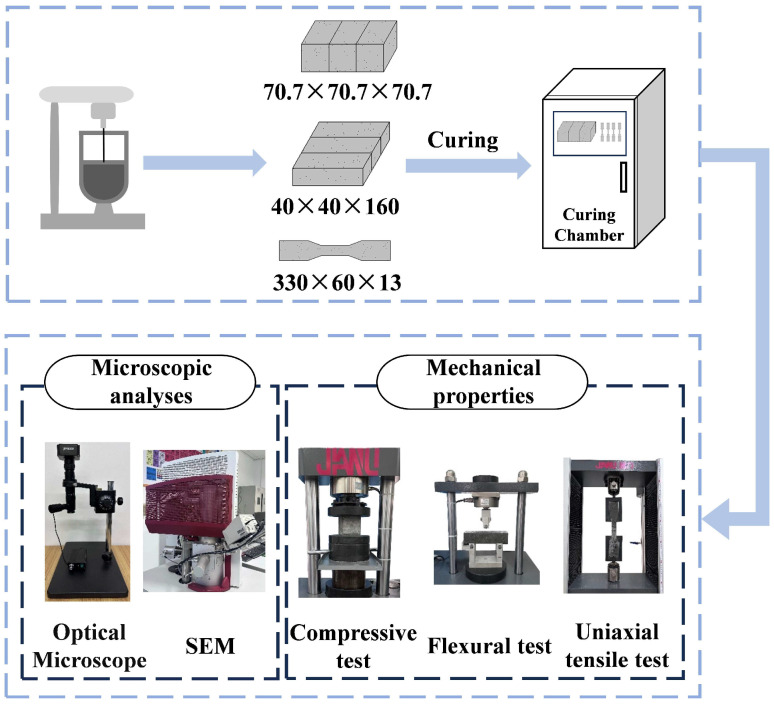
Flowchart of the experimental procedure.

Figure 3 illustrates the process of ECCs specimen preparation, curing, molding, and testing. First, the solid constituents, including cement, fly ash, and river sand, were mixed for 2 min using a mechanical mixer to ensure uniform blending. The superplasticizer was then dissolved in water to form a homogeneous solution, which was gradually added to the mixing container. After the mixture became uniform, fibers were slowly and manually added into the mortar to achieve better dispersion [37]. Finally, all constituents were mixed for an additional 5–8 min until a homogeneous concrete slurry was obtained. Specimens of different dimensions were prepared according to the requirements of various tests. The prepared mixture was poured into pre-prepared molds and vibrated for 1–2 min to ensure proper compaction. After 24 h, the specimens were demolded and subsequently cured for 28 days under standard conditions at a temperature of 20 ± 5 °C and a relative humidity of 95%.

#### 2.2.1. Compressive Test

In accordance with the testing method specified in JC/T 2461-2018 [38], cubic specimens measuring 70.7 × 70.7 × 70.7 mm at the appropriate age were removed from the curing box. Before testing, each specimen was placed on the lower platen of the compression testing machine and aligned vertically at the center between the upper and lower platens. Once the machine was activated and the upper platen approached the specimen, its position was adjusted to ensure uniform contact and pressure distribution. Loading was applied continuously and uniformly at a rate of 1.5 kN/s. The test automatically terminated when the specimen exhibited significant deformation and ultimately failed, with the failure load and the force–time relationship curve recorded during loading. The compressive strength of each mixture was determined as the arithmetic mean of three specimens.

Cubic compressive strength is calculated according to Equation (1):

(1) $$f_{cu}=\frac{F_{cu}}{A_{cu}}$$

where *f_cu_* is cubic compressive strength (MPa); *F_cu_* is the failure load of the specimen (N); *A_cu_* is the loaded area of the specimen (mm^2^).

#### 2.2.2. Flexural Test

In accordance with the test method specified in GB/T 17671-2021 [39], flexural strength tests were conducted using prismatic specimens with dimensions of 40 × 40 × 160 mm, specimens at the appropriate age were removed from the curing box and placed in the flexural testing machine. Loading was applied at a constant rate of 50 N/s until the specimen fractured, at which point the machine automatically stopped loading, the flexural strength was recorded. The average value of three specimens was taken as the flexural strength for each mix.

Flexural strength is calculated according to Equation (2):

(2) $$f_{f}=\frac{1.5F_{f}l}{b^{3}}$$

where *f_f_* is the flexural strength (MPa); *F_f_* is the failure load of the specimen (N); *l* is the span between supports (mm); *b* is the cross-sectional width of the specimen (mm).

#### 2.2.3. Uniaxial Tensile Test

**Figure 4:**
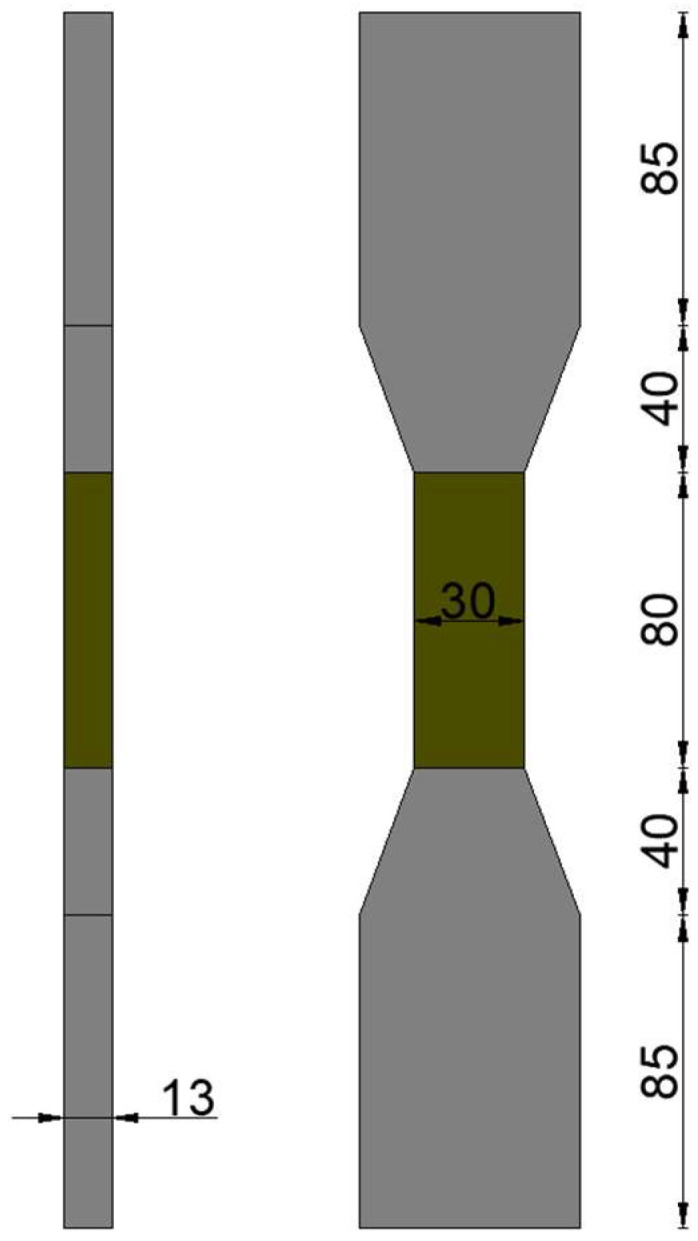
Dog-bone specimen dimensions (unit: mm).

In accordance with the test method specified in JC/T2461-2018 [38], the tensile properties of dog-bone-shaped specimens were evaluated using a direct tensile testing method. The dimensions of the test specimens were 330 mm × 60 mm × 13 mm, as shown in Figure 4. Before testing, the specimen was properly mounted and a gauge length of 80 mm was set. Prior to loading, careful alignment of the tensile apparatus was performed to avoid eccentric loading or uneven force distribution. Linear variable differential transformers (LVDTs) were installed on both sides of the specimen to synchronously record the force–displacement curves and measure elongation for the calculation of tensile strain. The loading rate for the tensile test was set at 0.5 mm/min.

## 3. Test Results and Analysis

In this section, the 28-day compressive, flexural, and uniaxial tensile test results of ECCs with different fiber types and contents are presented and analyzed in terms of strength, failure mode, and crack pattern. SEM observations of fracture surfaces and crack propagation are further conducted to elucidate the influence of fiber bridging and the fiber–matrix interface. These mechanical and microstructural results clarify the role of fiber characteristics in crack propagation and provide essential experimental input for the subsequent ANN-based tensile constitutive model.

### 3.1. Compressive Performance

#### 3.1.1. Compressive Destruction Form

ECCs cubic specimens exhibited excellent ductile failure characteristics under compressive test, which could be divided into four distinct stages: the elastic stage, microcrack initiation and propagation, macrocrack development, and final failure. At the initial loading stage, the specimen experienced linear elastic deformation. Owing to the high strength and modulus of the matrix and the relatively low applied load, no visible cracks appeared on the specimen surface. As the load increased, microcracks began to initiate and propagate in regions of stress concentration. During this phase, fibers effectively bridged the microcracks and inhibited their growth, thereby delaying the formation of macrocracks. When microcracks developed sufficiently and interconnected, macrocracks formed and extended through the specimen. At this stage, the bridging effect of the fibers gradually weakened, the matrix stiffness decreased significantly, and crack propagation accelerated. Once the external load exceeded the ultimate load-bearing capacity of the fibers or the fiber–matrix interface, the fibers failed through either pull-out or rupture, resulting in a rapid loss of load-carrying capacity and eventual specimen failure.

**Figure 5:**
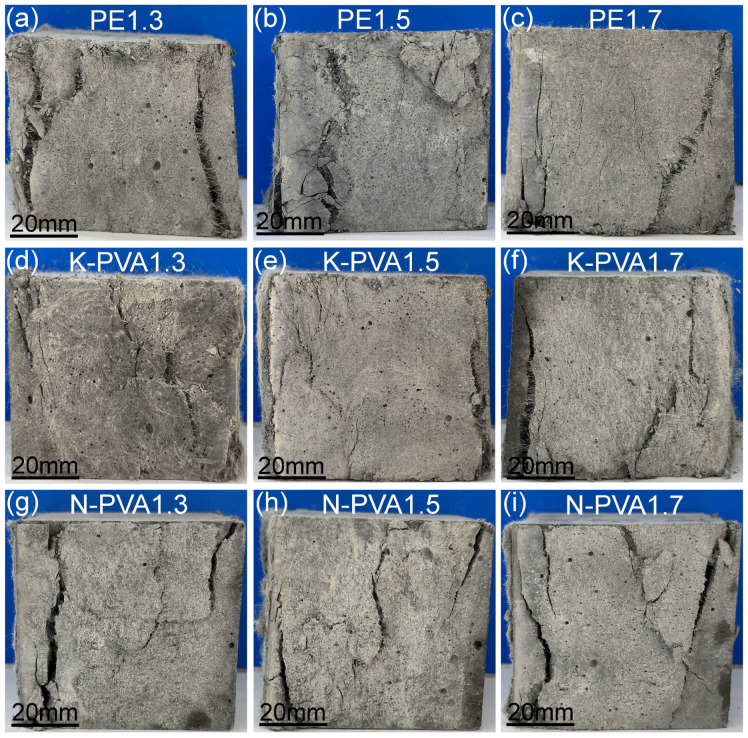
Compressive destruction form of ECCs specimens: (**a**) PE1.3; (**b**) PE1.5; (**c**) PE1.7; (**d**) K-PVA1.3; (**e**) K-PVA1.5; (**f**) K-PVA1.7; (**g**) N-PVA1.3; (**h**) N-PVA1.5; (**i**) N-PVA1.7.

The compressive destruction form of ECCs cubic specimens in each group are presented in Figure 5. As shown, when the PE fiber content was 1.3%, the specimens exhibited pronounced crack propagation and relatively wide macro-cracks, indicating compromised structural integrity. With an increase in fiber content to 1.5% and 1.7%, the bridging effect of the PE fibers was progressively enhanced, effectively mitigating crack development and improving the overall integrity of the specimens. Notably, at a fiber content of 1.7%, the specimens demonstrated the most favorable performance, characterized by significantly narrower cracks and a more stable destruction form, reflecting good crack resistance.

For specimens containing K-PVA fibers, at a fiber content of 1.3%, the specimen also exhibited pronounced cracking and inherently poor structural integrity. When the content was increased to 1.5%, crack propagation was effectively mitigated, and the specimens exhibited enhanced densification and overall structural integrity. However, at a fiber content of 1.7%, although crack propagation was partially controlled, the specimen still exhibited failure characteristics indicative of brittleness, suggesting that an excessively high fiber content can impair the interfacial bonding between the fibers and the cementitious matrix [19].

For specimens incorporating N-PVA fibers, a fiber content of 1.3% led to wide and unevenly distributed cracks, reflecting poor structural integrity. When the fiber content was increased to 1.5%, both the number and width of cracks were reduced, significantly improving the overall performance of the specimens. This indicates that the bridging effect of the fibers was effectively exerted at this content, effectively suppressing crack propagation. However, at a fiber content of 1.7%, the crack-control performance declined, with accelerated crack development and more prominent macro-cracks, resulting in inferior overall integrity. This phenomenon suggests that excessive fiber content can reduce the homogeneity of the matrix, causing localized crack concentration and ultimately diminishing the compressive strength of the specimens [40].

#### 3.1.2. Compressive Strength

**Figure 6:**
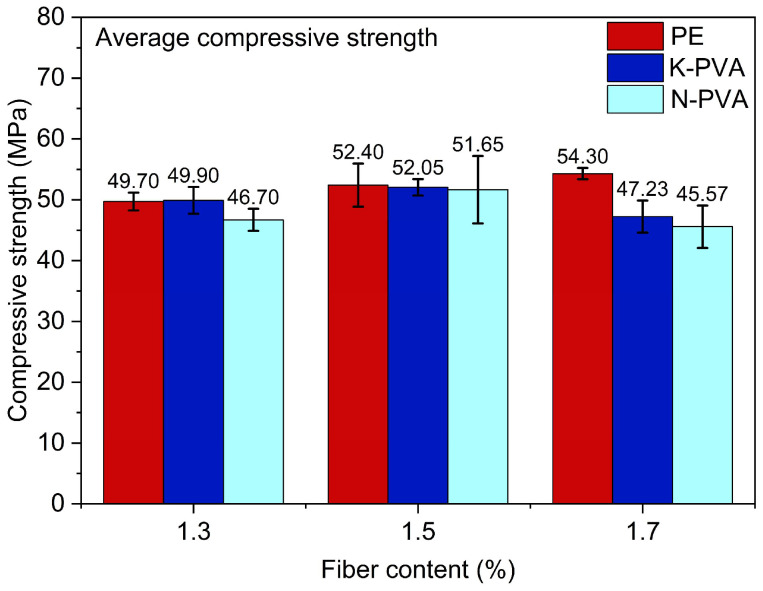
Compressive strength of ECCs specimens.

The compressive strength results of the ECCs specimens are shown in Figure 6. The influence of the fiber types (PE fibers, K-PVA fibers, and N-PVA fibers) on the compressive performance of ECCs exhibited a characterization of nonlinear changes. Test results indicate that the compressive strength of specimens incorporating PE fibers increases steadily with fiber content, rising from 49.70 MPa at 1.3% to 54.30 MPa at 1.7%. This corresponds to improvements of approximately 5.43% (PE1.5) and 9.26% (PE1.7) relative to PE1.3. This enhancement is mainly attributed to the high elastic modulus and tensile strength of PE fibers [41], as well as their excellent dispersibility [42], which enable them to effectively bridge cracks within the matrix, thereby suppressing microcrack propagation and enhancing the overall load-bearing capacity.

In comparison, the compressive strength of ECCs reinforced with K-PVA fibers increased from 49.90 MPa at 1.3% fiber content to 52.05 MPa at 1.5%, corresponding to an increase of approximately 4.31%. However, when the content was further raised to 1.7%, the strength dropped to 47.23 MPa, about 5.35% lower than that of K-PVA1.3. This suggests that an excessive fiber content can result in fiber agglomeration and increased porosity, which in turn disrupts matrix compactness [43] and impair the reinforcing effect.

The compressive performance of ECCs incorporating N-PVA fibers showed a more pronounced sensitivity to fiber content. When the content increased from 1.3% to 1.5%, the compressive strength rose markedly from 46.70 MPa to 51.65 MPa, representing a growth of approximately 10.60%, which indicates a notable reinforcing effect. However, further increasing the fiber content to 1.7% led to a substantial reduction in strength, dropping to 45.57 MPa, around 2.42% lower than that of N-PVA1.3. This behavior suggests that, owing to the relatively poor dispersibility of N-PVA fibers, excessive fiber content can induce agglomeration, thereby disrupting the matrix continuity and increasing the internal porosity [17]. In addition, the swelling caused by water absorption at high fiber content could result in stress concentrations within the interfacial transition zone (ITZ), thereby weakening the overall compressive strength [44].

Cross-sectional comparison indicates that at a fiber content of 1.3%, the compressive strength of specimens containing PE fibers is slightly lower than that of those reinforced with K-PVA fibers. However, with increasing fiber content, the compressive strength of PE fiber-reinforced specimens demonstrates a more consistent upward trend. At higher fiber content, the compressive strength of PE fiber-reinforced specimens is clearly superior to that of specimens incorporating both types of PVA fibers. This further substantiates the comprehensive advantages of PE fibers in terms of dispersibility, mechanical properties, and their synergistic interaction with the cementitious matrix [41,42].

### 3.2. Flexural Performance

#### 3.2.1. Flexural Destruction Form

In the three-point bending test, ECCs specimens demonstrated a failure process distinctly different from that of conventional concrete, characterized by pseudo-strain hardening behavior. during the initial loading phase, the specimens underwent elastic deformation, with mid-span deflection increasing linearly with the applied load and no visible surface cracks observed. As the load approached its peak, localized stress concentrations developed in the tensile zone at the bottom of the specimen. However, constrained by fiber bridging, no macrocrack propagation occurred. Upon reaching the ultimate load, the load–deflection curve dropped sharply, indicating brittle fracture of the matrix, and the specimen failed. However, due to the bridging action of fibers across the fracture surface [45,46], it did not completely separate. Even after final failure, the specimen retained a relatively high degree of structural integrity.

**Figure 7:**
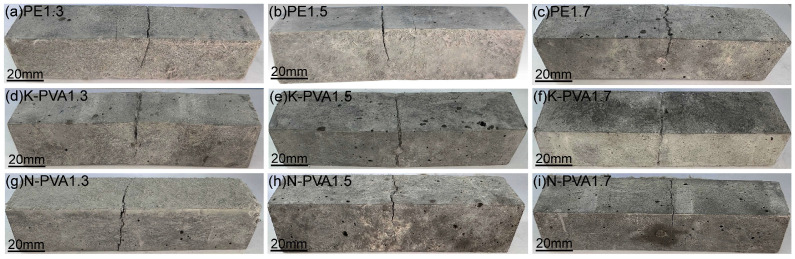
Flexural destruction form of ECCs specimens: (**a**) PE1.3; (**b**) PE1.5; (**c**) PE1.7; (**d**) K-PVA1.3; (**e**) K-PVA1.5; (**f**) K-PVA1.7; (**g**) N-PVA1.3; (**h**) N-PVA1.5; (**i**) N-PVA1.7.

Figure 7 illustrates the flexural destruction form of ECCs specimens under different fiber types and content. As shown, both the fiber types and content have a significant influence on crack propagation and fracture behavior of the specimens.

For the specimens incorporating PE fibers (Figure 7a–c), a dominant through-thickness crack with a relatively large width was observed at a fiber content of 1.3%, accompanied by a small number of randomly distributed secondary cracks. As the fiber content increased to 1.5% and 1.7%, pronounced multiple cracking appeared on the specimen surfaces. Several fine cracks were distributed on both sides of the main crack, and the crack spacing was significantly reduced, indicating that the excellent bridging effect of PE fibers at higher content can effectively enhance the integrity of the fracture section.

For the specimens incorporating K-PVA fibers (Figure 7d–f), at a fiber content of 1.3%, the cracking was also dominated by main cracks, which exhibited a high degree of penetration and relatively large spacing. As the fiber content increased to 1.5% and 1.7%, the width of the primary cracks was notably reduced, and the fibers formed effective bridging connections near the crack surfaces. Nevertheless, localized fiber agglomeration was observed in some regions.

In comparison, for the specimens incorporating K-PVA fibers (Figure 7g–i), the cracking behavior was predominantly characterized by main cracks at different fiber content, with a high degree of crack penetration, and the fibers exhibited a relatively weak ability to inhibit crack propagation. Although the width of the main cracks was slightly reduced at fiber content of 1.5% and 1.7%, but no pronounced multiple cracking behavior was observed, and crack branching was only visible in localized areas, indicating that this type of fiber still suffers from insufficient dispersion and limited bridging performance at high content.

#### 3.2.2. Flexural Strength

**Figure 8:**
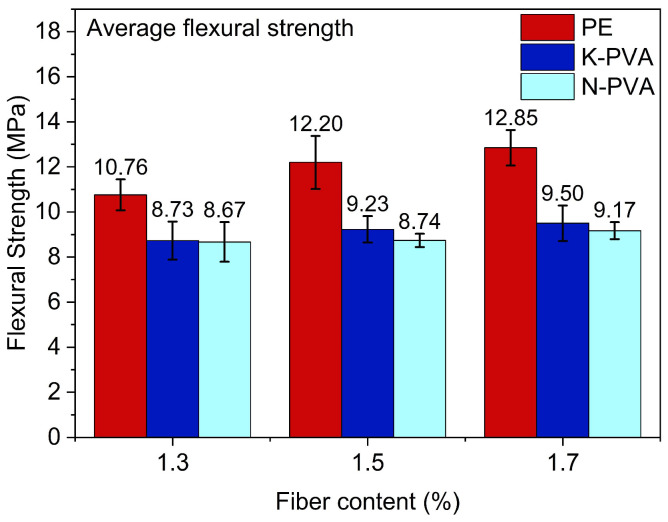
Flexural strength of ECCs specimens.

**Figure 9:**
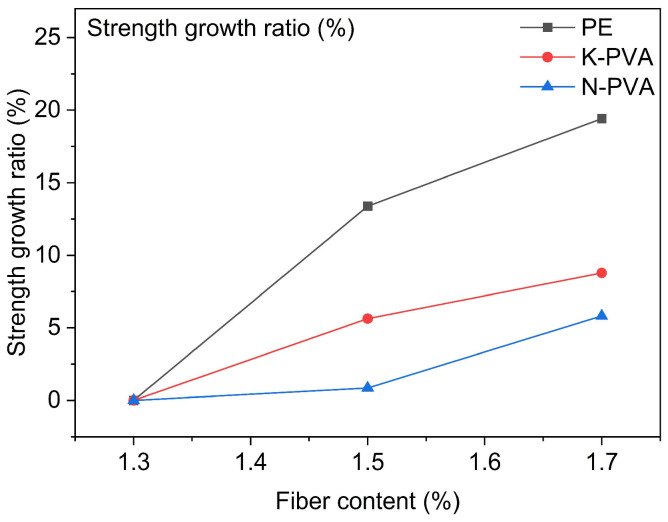
Growth ratio of flexural strength in ECCs specimens.

With different fiber types and different fiber content, the flexural strength test results of ECCs are presented in Figure 8, and the strength growth ratio of ECCs specimens with different fiber types, relative to their respective 1.3% fiber content groups, are shown in Figure 9.

As shown in Figure 8, with the increase in fiber content, the flexural strength of the specimens generally showed an upward trend; however, the reinforcing effects varied across different fiber types: for specimens incorporating PE fibers, increasing the content from 1.3% to 1.5% and 1.7% led to flexural strength increases of approximately 13.38% and 19.42%, respectively, demonstrating a notably strong reinforcing effect. In comparison, specimens incorporating K-PVA fibers exhibited flexural strength increases of approximately 5.63% and 8.78% at the same fiber content, while those with N-PVA fibers showed more limited improvements of around 0.85% and 5.82%, respectively. These results indicated that PE fibers demonstrated a more pronounced effect in enhancing the flexural performance of ECCs [47,48], primarily due to their superior mechanical properties. However, in PVA fibers, especially N-PVA fibers, which exhibited relatively weaker enhancement in ECCs flexural performance due to inherent limitations in their physical properties and interfacial bonding characteristics [49]. Furthermore, increasing fiber content did not necessarily result in a linear improvement in flexural strength, as excessive content could lead to issues such as poor dispersion or weak interfacial bonding with the matrix [20], thereby compromising the overall performance.

### 3.3. Uniaxial Tensile Performance

#### 3.3.1. Tensile Destruction Form

Due to the incorporation of fibers, the specimens of ECCs did not fail immediately under tensile loading. Instead, it exhibited multiple cracking behavior. The tensile destruction form of ECCs progressed through three distinct stages: the elastic stage, the strain-hardening stage, and the strain-softening stage.

First of all, in the elastic stage, the stress–strain was a linear relationship, and the specimen surface did not appear cracks. At this stage, the tensile load was primarily borne by the matrix. As the tensile load continued, the first microcrack formed at the weakest point of the specimen, accompanied by a slight drop in stress. At this time, the elastic stage was ending, and immediately entered into the second stage, that was the strain-hardening phase.

After the formation of the first microcrack, the specimen surface entered a multiple cracking mode. During this phase, the tensile load continued to increase, and the fibers interacted with the cementitious matrix. Audible cracking sounds were emitted from the matrix, and a large number of microcracks appeared near the midsection of the specimen. During this period, the fibers exerted their intrinsic bridging effect to inhibit further crack propagation. The strain-hardening phase continued until no new cracks formed, while the existing ones further extended and their widths increased, ultimately reaching a saturated crack distribution.

**Figure 10:**
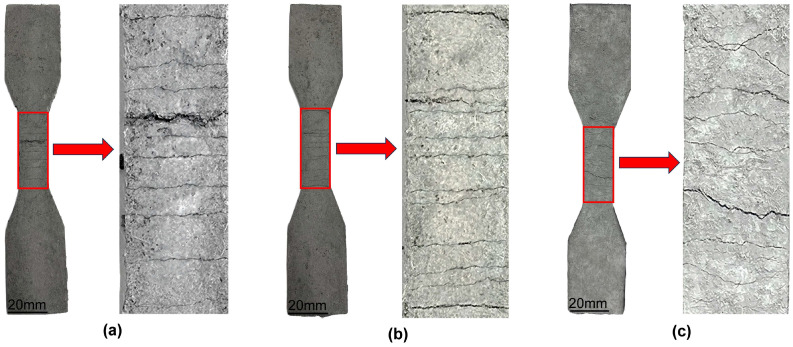
Crack destruction form of ECCs specimens: (**a**) N-PVA; (**b**) K-PVA; (**c**) PE.

Next, the third stage, known as the strain-softening phase. In this stage, the specimen reached its ultimate load and loading continued, the crack-bridging capacity of the fibers gradually deteriorates. One or more microcracks progressively widened and developed into dominant cracks, eventually, the specimen could no longer sustain the load and failed through fracture along the major crack. The uniaxial tensile destruction form of ECCs are illustrated in Figure 10.

**Figure 11:**
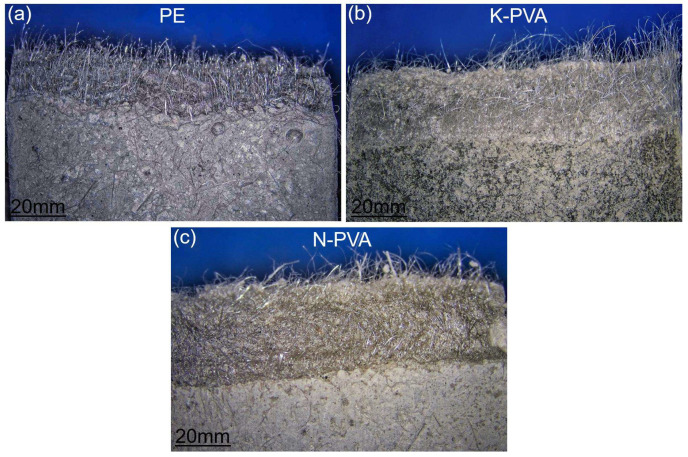
Fracture surface morphology of different fiber types.

Figure 11 shows the fracture surface morphology of the fiber–matrix interface after specimen failure. As shown in Figure 11a, the fracture surface of the specimen incorporating PE fibers exhibited a large number of fibers pulled out from the matrix, exhibiting a relatively uniform distribution with smooth surfaces and no obvious signs of tensile deformation or fiber rupture. As shown in Figure 11b, the fracture surface of the specimen incorporating K-PVA fibers revealed relatively rough fiber surfaces, with some fibers exhibiting signs of splitting, indicating that plastic deformation occurred during the tensile process. In comparison to PE fibers and K-PVA fibers, the fracture surface of specimens incorporating N-PVA fibers exhibited markedly non-uniform fiber distribution, some fibers appeared bent or adhered to the fracture interface, as shown in Figure 11c.

#### 3.3.2. SEM Analysis

**Figure 12:**
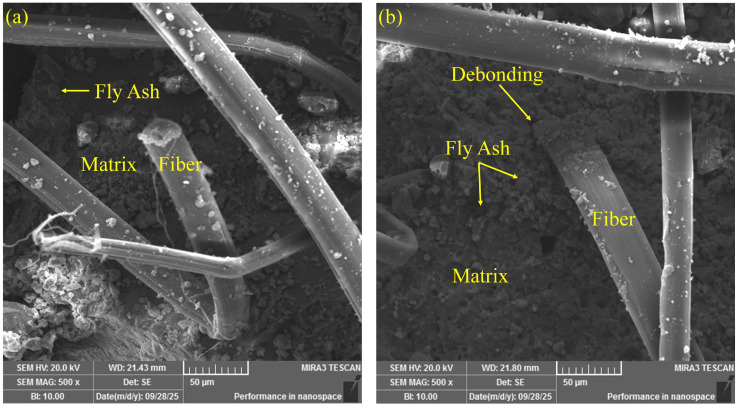
Microscopic morphology of fracture surfaces in PE-ECCs specimens: (**a**) fibers embedded in the matrix with fly ash particles; (**b**) fiber–matrix debonding.

**Figure 13:**
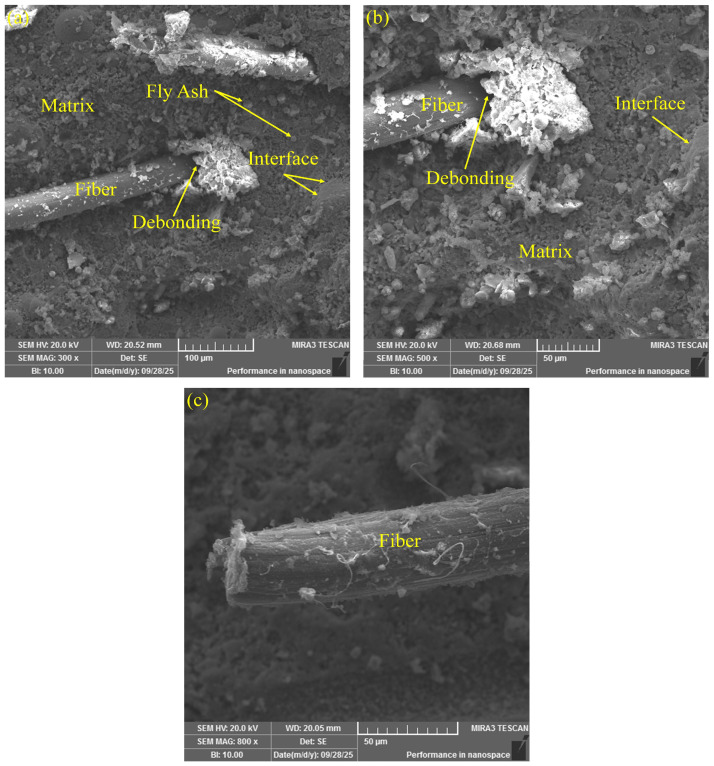
Microscopic morphology of fracture surfaces in K-PVA-ECCs specimens: (**a**,**b**) fiber–matrix debonding in the interfacial transition zone; (**c**) K-PVA fibers surface.

**Figure 14:**
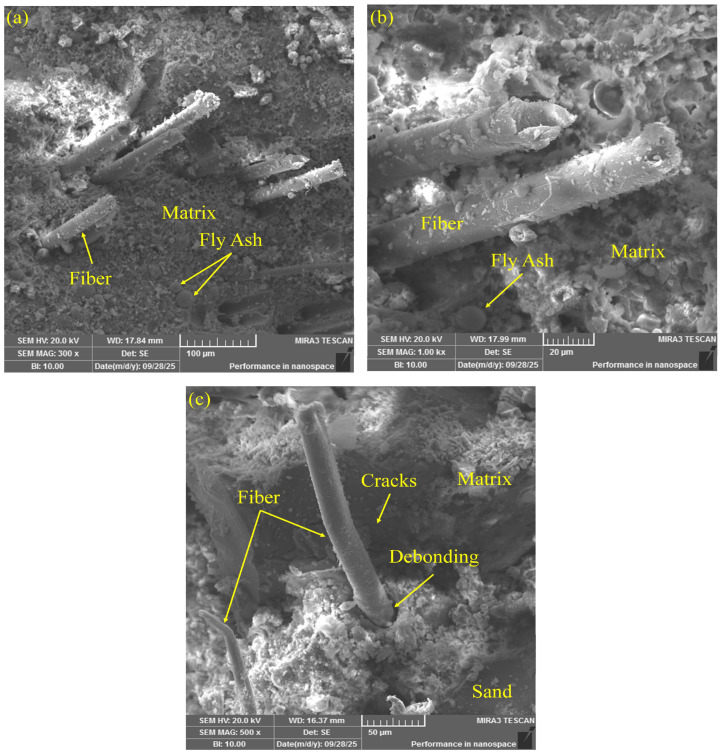
Microscopic morphology of fracture surfaces in N-PVA-ECCs specimens: (**a**) fibers, matrix and fly ash particles in the interfacial transition zone; (**b**) magnified view of (**a**); (**c**) fiber–matrix debonding and crack propagation near sand particles.

To systematically investigate the effects of different fiber types on the interfacial structure and failure mechanisms of ECCs materials, scanning electron microscopy (SEM) was employed to examine the microstructural morphology of the fracture interfaces. Figure 12, Figure 13 and Figure 14 present the fracture interface characteristics of ECCs specimens reinforced with PE fibers, K-PVA fibers, and N-PVA fibers, respectively. By comparatively analyzing the distribution patterns of various fibers within the fracture zone, the structure of the ITZ, the surface morphology of the fibers, and their bonding characteristics with the matrix, this study provides in-depth insights into the mechanisms by which different fiber systems influence crack propagation behavior and energy dissipation pathways. The observed differences in microstructural features significantly affect the fiber bridging capacity and the strain-hardening behavior of the composites, providing a microstructural foundation for the subsequent development and performance optimization of the ANN-based constitutive prediction model.

Figure 12a, b present the fracture surface morphology of ECCs specimens reinforced with PE fibers. It was observed that the PE fibers exhibited a generally smooth surface in the fracture region, lacked a distinct layer of hydration products, and showed visible gaps and continuous slip marks at certain interfacial areas. These morphological features indicate a weak interfacial bonding between the PE fibers and the cementitious matrix. This interfacial behavior is primarily attributed to the hydrophobic nature and low surface energy of PE fibers [12,50], which prevent the formation of strong chemical bonds with the cement paste [51]. Consequently, during tensile loading, fiber pull-out becomes the dominant failure mode [52], rather than fiber rupture, with energy dissipation mainly occurring through the pull-out process [53]. Furthermore, a significant number of small, spherical fly ash particles were distributed within the ITZ. These particles helped fill the matrix pores and improve the compactness of the interfacial structure.

Figure 13a–c illustrate the fracture surface morphology of ECCs specimens reinforced with K-PVA fibers. As observed in the images, the fiber–matrix interfaces exhibited strong bonding, with substantial amounts of hydration products adhered to the fiber surfaces. The fibers displayed a relatively rough texture, which suggested pronounced interfacial interactions between the K-PVA fibers and the cementitious matrix during the fracture process. Furthermore, edge tearing was observed on some fibers, suggesting that under large strain conditions, the fibers underwent nonlinear failure induced by tensile stress.

This interfacial morphology indicates a relatively strong bond between the K-PVA fibers and the cementitious matrix. Owing to their specially treated surfaces, K-PVA fibers exhibit favorable hydrophilicity and interfacial bonding properties [6]. During the tensile process, the fibers effectively engage with the matrix to resist crack propagation. However, under higher stress conditions, the fibers were found to be prone to rupture [24,54], with energy dissipation primarily occurring through a synergistic mechanism of fiber pull-out and rupture [24].

Figure 14a–c illustrate the fracture surface morphologies of ECCs specimens reinforced with N-PVA fibers. The distribution of hydration products on the fiber surfaces was uneven, and distinct signs of fiber–matrix separation were evident in certain interfacial regions. Moreover, numerous surface pores and microcracks were observed, along with localized fiber agglomeration. These interfacial features indicate a relatively weak bond between the fibers and the cementitious matrix, which hinders the fibers’ ability to effectively bridge cracks during tensile loading [16]. In addition, the poor dispersibility of N-PVA fibers resulted in non-uniform fiber distribution within the matrix [55], with failure predominantly occurring through fiber rupture [56].

#### 3.3.3. Tensile Stress–Strain Curve

**Figure 15:**
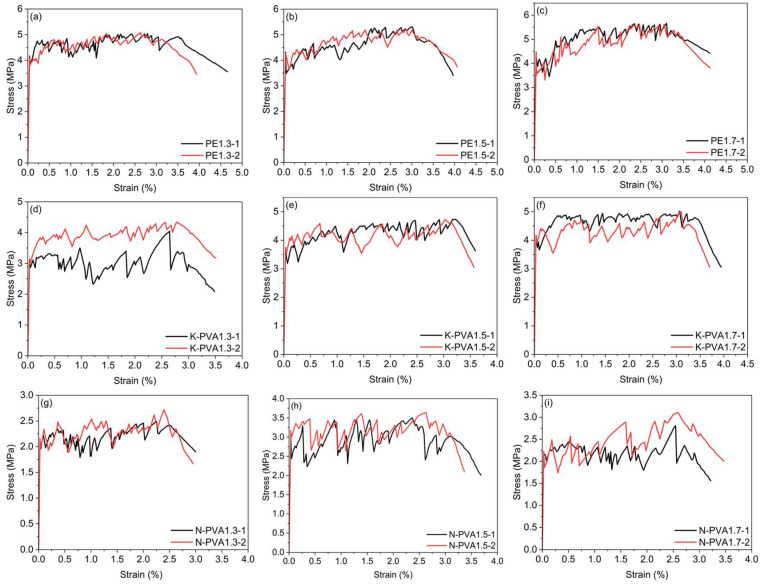
Stress–strain curves of ECCs specimens group: (**a**) PE1.3; (**b**) PE1.5; (**c**) PE1.7; (**d**) K-PVA1.3; (**e**) K-PVA1.5; (**f**) K-PVA1.7; (**g**) N-PVA1.3; (**h**) N-PVA1.5; (**i**) N-PVA1.7.

**Figure 16:**
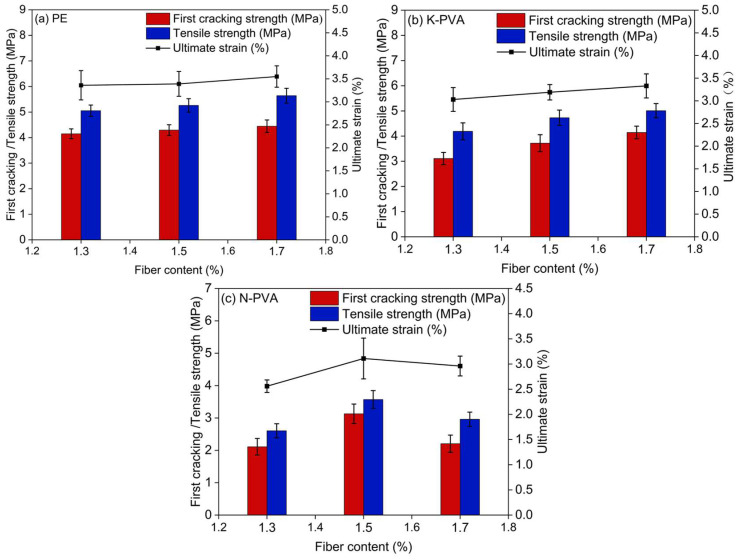
Comparison of tensile performance parameters of ECCs specimens group.

The tensile stress–strain curves of the ECCs specimens group are presented in Figure 15. Figure 16 illustrates the comparison between different fiber contents and the first cracking strength, tensile strength, and ultimate strain for each fiber type, and the corresponding experimental parameters are summarized in Table 4. As shown in Figure 15, for ECCs specimens incorporating the same type of fibers, as the fiber content increased, the stress–strain curves of the specimens with PE fiber and K-PVA fiber both exhibited an upward trend in strength. In contrast, for specimens with N-PVA fibers, the highest strength were observed at a fiber content of 1.5%; further increasing the fiber content resulted in a decrease in strength. This indicates that both PE fibers and K-PVA fibers exhibit good dispersibility with increasing fiber content, and are capable of effectively bridging cracks and transferring stress [57], thereby enhancing the strength of ECCs. On the other hand, when N-PVA fibers are used, several limitations arise. First, due to insufficient surface treatment, the interfacial bond strength between the fiber and the matrix is relatively weak [16]. When the fiber content becomes excessively high, poor dispersibility can lead to fiber agglomeration and uneven distribution [55], which in turn forms weak zones that act as stress concentration zones. Second, N-PVA fibers possess a comparatively lower elastic modulus [15], making them less effective at stress transfer under high content, which eventually results in failure of the cementitious matrix.

The stress–strain curves of ECCs specimens exhibited a progressively decreasing strength trend from those reinforced with PE fibers, to those with K-PVA fibers, and finally to those with N-PVA fibers. This variation is primarily attributed to differences in the mechanical properties of the fibers and their interfacial interaction mechanisms. PE fibers possess a high elastic modulus and tensile strength, enabling them to carry part of the tensile load during crack initiation and propagation [41]. Moreover, due to their low surface energy, PE fibers exhibit weak interfacial bonding with the matrix [50], and primarily dissipate energy through fiber pull-out rather than rupture during tension. This pull-out-dominated mechanism contributes to the stable propagation of cracks and energy dissipation, thereby enhancing the ductility and overall strength of ECCs materials [52].

In contrast, although K-PVA fibers exhibit stronger interfacial bonding capacity and can be effectively anchored within the cementitious matrix, they are more prone to tensile rupture under high-stress conditions [54], which results in a restricted pathway for energy dissipation. Additionally, K-PVA fibers possess a relatively uniform diameter distribution [58] and are treated with a nanoscale hydrophilic coating on the surface [24], which further enhances their chemical bonding with cement hydration products [59], thereby improving their mechanical properties and stability. As a result, compared to N-PVA fibers, K-PVA fibers demonstrate a more pronounced effect in enhancing the strength of ECCs.

N-PVA fibers exhibit relatively weaker dispersion, interfacial bonding ability, and mechanical properties due to limitations in their manufacturing process and surface treatment [55,60], which results in a reinforcing effect that is weaker than that of PE fibers and K-PVA fibers.

As shown in Figure 16 and Table 4, the tensile performance of ECCs varies significantly with fiber type and content. For PE fibers, both the first cracking strength (σ_cr_) and tensile strength (σ_p_) consistently increased within the fiber content range of 1.3% to 1.7%. Compared to PE1.3, the first cracking strength values of PE1.5 and PE1.7 increased by approximately 3.61% and 7.23%, respectively, while the tensile strength increased by about 3.95% and 11.46%. The ultimate strain (ε_tu_) also rose correspondingly.

For K-PVA fibers, the overall mechanical properties remained favorable across the 1.3–1.7% content range. The first cracking strength increased from 3.11 MPa to 4.15 MPa, and the tensile strength increased from 4.19 MPa to 5.01 MPa. As the fiber content increased, the first cracking strength improved by approximately 19.61% and 33.44%, and the tensile strength increased by 12.89% and 19.57%, respectively. The ultimate strain also steadily increased, indicating strong crack-bridging capacity and good ductility [8].

For N-PVA fibers, the best tensile performance was observed at a fiber content of 1.5%. When the content exceeded this level, fiber agglomeration resulted in poor dispersion, leading to significant decreases in tensile strength and ultimate strain [17,55]. Specifically, as the content increased from 1.5% to 1.7%, the first cracking strength and tensile strength dropped by approximately 29.39% and 17.09%, respectively.

## 4. ANN-Based Prediction of the Tensile Constitutive Model of ECCs

### 4.1. Model Establishment

In current cutting-edge research, two simplified forms of tensile constitutive model for ECCs have been predominantly established: the bilinear model [61] and the trilinear model [62,63]. The trilinear model can be further classified into two distinct categories based on whether the strain-softening stage is considered: one that includes the strain-softening stage, and one that excludes it. However, analyses of numerous engineering cases, along with corresponding experimental data, have revealed that the strain-softening stage faces significant limitations in practical engineering applications, making it difficult to be fully and effectively utilized. This is mainly attributed to the complexity of practical engineering environments, as well as the limitations of current construction technologies and monitoring methods at this stage.

Based on a thorough analysis of the fundamental characteristics of the tensile stress–strain response of ECCs, and with comprehensive consideration of key multidimensional factors such as construction feasibility and the complexity of acquiring model parameters in engineering applications, a number of prominent scholars, including Tetsushi Kanda [61] and Li et al. [64], through rigorous theoretical derivations and extensive experimental verification, recommended the bilinear model as a more advantageous option for constructing the tensile constitutive model of ECCs. However, the bilinear model also has certain limitations: it approximates the strain-hardening stage with a constant slope in the tensile constitutive law, making it difficult to accurately capture the characteristics of this stage. Therefore, in this study it is used for engineering simplification and parametric representation, rather than as a replacement for more refined nonlinear constitutive models.

**Figure 17:**
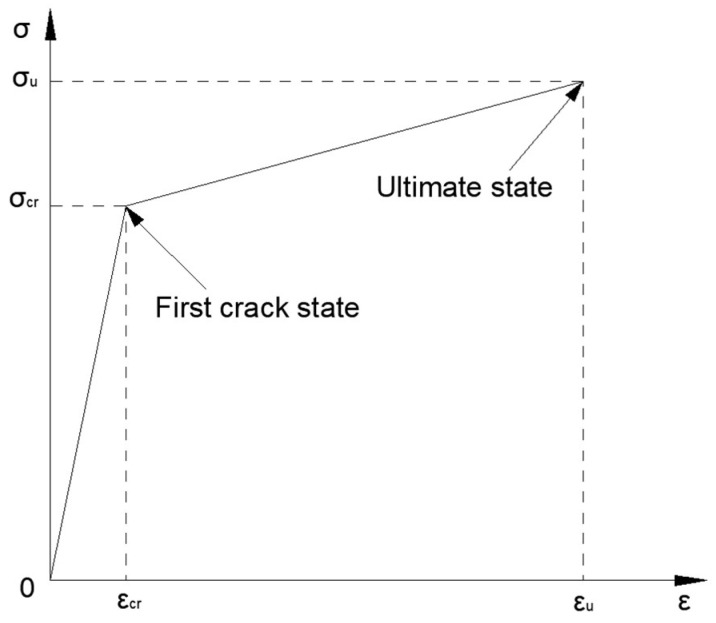
Bilinear model.

The complex stress–strain curves obtained from the experiments were simplified using an idealized bilinear tensile constitutive model for ECCs [61], as shown in Figure 17. This curve can be fully characterized by two coordinate points, which correspond to four output parameters: first cracking strain, first cracking stress, ultimate strain, and ultimate stress. These parameters were predicted by the ANN model.

**Figure 18:**
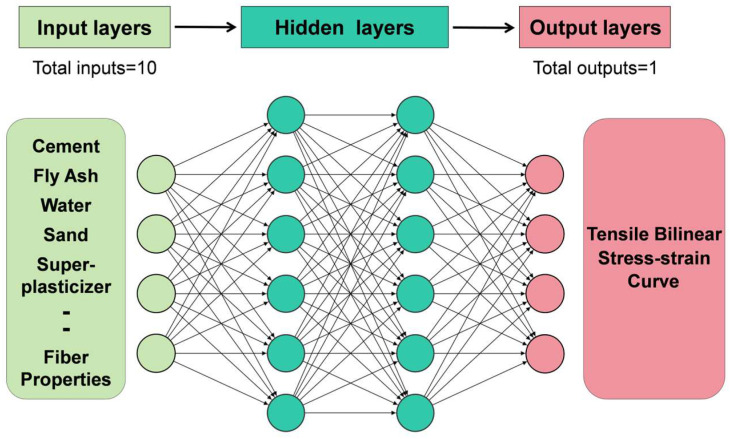
Schematic diagram of the ANN model workflow.

Artificial Neural Network (ANN) technology has been widely recognized for its high accuracy and effectiveness, owing to its ability to perform parallel data processing through interconnected neurons, thereby significantly improving computational speed and efficiency [65]. An ANN typically consists of three fundamental layers: the input layers, the hidden layers, and the output layers. While the structures of the input and output layers generally remain fixed, the hidden layers are adaptable components whose number and configuration are dynamically adjusted in accordance with the complexity of the modeled system. The architecture of the hidden layers plays a pivotal role in determining the model’s performance. Appropriately increasing the number of hidden layers can enhance the model’s fitting capability and reduce prediction errors. However, an excessive number of hidden layers can lead to overfitting, where the model exhibits excellent performance on training data but fails to generalize well on the testing and validation datasets [66]. The workflow of the ANN model constructed in this study is illustrated in Figure 18.

In this study, four independent deep neural network models were constructed, each corresponding to one of the four key tensile performance parameters (first cracking strain, first cracking stress, ultimate strain, and ultimate stress). All models adopt a multilayer perceptron architecture with residual connections, in order to enhance gradient propagation in deeper networks and mitigate degradation. The network consists of an input mapping layer, two hidden layers (residual module), and an output layer. The input mapping layer is used to project the original input features into a high-dimensional space; the residual module is composed of two fully connected layers, each equipped with a GELU activation function and batch normalization, while retaining residual connections from the module input to strengthen feature representation; the output layer performs single-output regression prediction. All networks use the He initialization strategy to ensure stable gradient propagation, and the Levenberg–Marquardt optimization algorithm is employed to improve convergence efficiency. To prevent overfitting, early stopping is applied during training by monitoring the performance on the test set, and, together with batch normalization, this enhances the generalization capability of the models.

### 4.2. Dataset

#### 4.2.1. Data Collection

First, to develop a predictive model, it is essential that comprehensive and representative data be collected to fully capture the key characteristics of the system being modeled. In this study, the dataset was compiled from published literature sources [4,37,67,68,69,70,71,72,73,74,75,76,77,78,79,80,81,82,83,84,85,86,87,88,89,90,91,92,93,94,95,96,97,98,99,100,101,102,103,104,105,106,107] together with a portion of experimental data, totaling 167 valid data groups. Each dataset comprises 14 attributes, including 10 input parameters and 4 output parameters. Table 5 provides a detailed statistical overview of the dataset used in this study. The dataset encompasses three fiber types, the specific characteristics of which are presented in Table 6.

**Figure 19:**
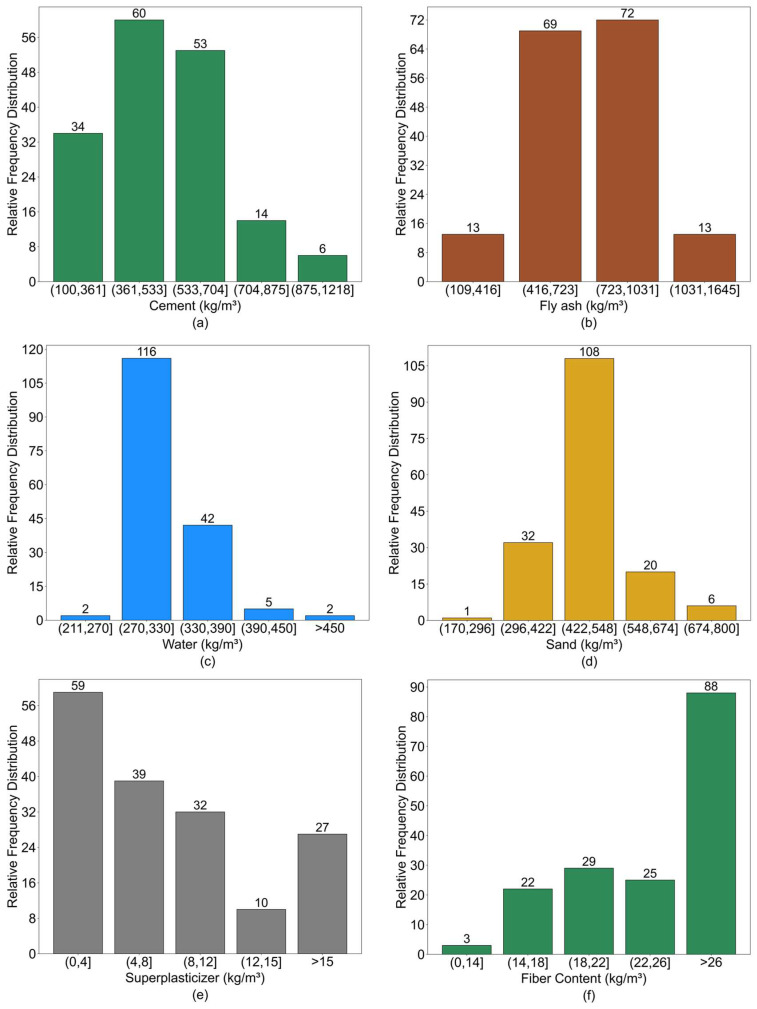

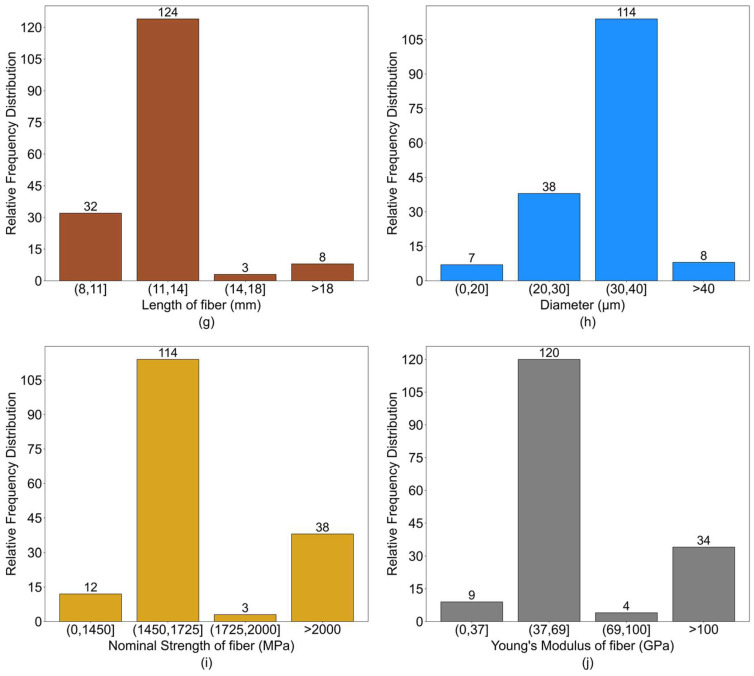
Histogram of data distribution: (**a**) Cement; (**b**) Fly ash; (**c**) Water; (**d**) Sand; (**e**) Superplasticizer; (**f**) Fiber Content; (**g**) Length of fiber; (**h**) Diameter; (**i**) Nominal Strength of fiber; (**j**) Young’s Modulus of fiber.

To ensure the comprehensiveness of the model, the input parameters were classified into two major categories: matrix composition and fiber characteristics. The matrix composition includes cement, fly ash, sand, water, and superplasticizer, while the fiber characteristics cover fiber content, length, diameter, tensile strength, and elastic modulus. The distribution characteristics of the dataset are visually illustrated using the histograms presented in Figure 19.

#### 4.2.2. Data Preprocessing and Normalization

**Figure 20:**
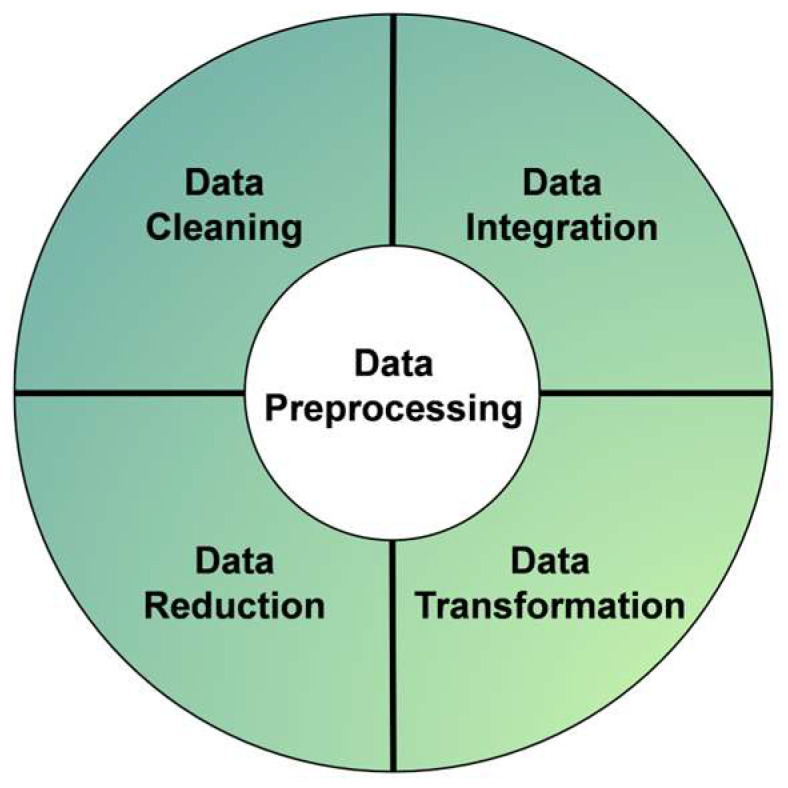
Process of raw data processing.

During the ANN modeling process, the quality of the data and the methods used to handle it directly influence and determine the reliability and accuracy of the model’s predictions. Therefore, before establishing the ANN prediction model, it is essential to preprocess and normalize the raw data. This typically involves four main steps: data cleaning, data transformation, data reduction, and data integration [108], as shown in Figure 20.

Initially, data cleaning is performed to identify and correct erroneous entries and outliers in the dataset, thereby reducing the impact of abnormal data on the model’s predictive accuracy. In this study, the data cleaning process entailed removing four data points with excessively high tensile strength, specifically those above 10 MPa. Next, data transformation is conducted by converting each stress–strain curve into four key parameters of an idealized bilinear model: first-cracking strain, first-cracking stress, ultimate strain, and ultimate stress. Additionally, data simplification and integration are implemented to further refine the dataset for efficient model training.

**Figure 21:**
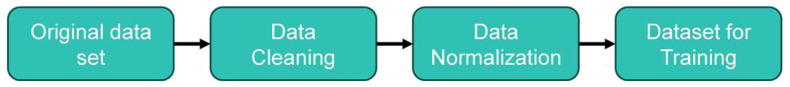
Preprocessing workflow.

After completing the data preprocessing steps, dataset normalization was carried out. Since the input parameters involve different physical quantities, such as length, diameter, and strength—with markedly different numerical ranges, normalization was applied in this study to rescale all values to the range of 0 to 1. This normalization process significantly improved numerical stability during model training and enhanced the predictive accuracy of the model. The overall preprocessing workflow employed in this study is illustrated in Figure 21.

### 4.3. Training and Performance Evaluation of the ANN Model

#### 4.3.1. Training Method and Hyperparameter Optimization of the ANN Model

To ensure the stability and accuracy of the ANN-based predictive model, this study conducted a systematic adjustment and optimization of hyperparameters and employed a suitable training strategy. Hyperparameters, which are external parameters that define and govern the learning process, play a pivotal role in influencing the model’s fitting performance and predictive accuracy [109]. Inappropriate hyperparameter selection can result in under-fitting or over-fitting, thereby affecting the accuracy of prediction outcomes [110].

For the training methodology, the Levenberg–Marquardt (LM) optimization algorithm [111] was adopted in this study. This algorithm integrates the strengths of both gradient descent and Gauss–Newton methods, providing efficient convergence and high predictive accuracy, particularly for small- and medium-scale datasets.

During the hyperparameter tuning process, a systematic evaluation was conducted using sensitivity analysis and iterative optimization to assess the impact of different hyperparameters on prediction accuracy. The results demonstrated that the number of neurons in the hidden layer was the most critical factor influencing prediction performance. When the number of neurons was too small, the model failed to adequately capture the complex nonlinear relationships between input and output variables. Conversely, When the number of neurons increased within a certain range, the model tended to become over-fitting. Based on multiple rounds of comparative training and testing, this study selected the neuron configuration that achieved the best balance between fitting performance on the training set and predictive performance on the test set. The optimal hyperparameters adopted in this study are summarized in Table 7.

#### 4.3.2. Evaluation of Model Performance Indicators

To comprehensively evaluate the accuracy of the ANN model’s performance, four fundamental statistical metrics were employed in this study: root mean square error (RMSE), mean absolute error (MAE), Pearson correlation coefficient (R), and coefficient of determination (R^2^). These indicators help establish a quantitative relationship between the predicted (*Ypre*) and the actual (*Yactual*) results.

(3) $$RMSE=\sqrt{\frac{\sum_{i=1}^{n}{\left(Y_{pre}-Y_{actual}\right)}^{2}}{n}}$$

(4) $$MAE=\sum_{i=1}^{n}\frac{\left|Y_{pre}-\left.Y_{actual}\right|\right.}{n}$$

(5) $$R^{2}=\frac{\sum_{i=1}^{n}{\left(Y_{pre}-\bar{Y_{pre}}\right)}^{2}}{\sum_{i=1}^{n}{\left(Y_{actual}-\bar{Y_{actual}}\right)}^{2}}$$

(6) $$R=\frac{\sum_{i=1}^{n}\left(Y_{pre}-\bar{Y_{pre}}\right)\left(Y_{actual}-\bar{Y_{actual}}\right)}{\sqrt{\sum_{i=1}^{n}{\left(Y_{pre}-\bar{Y_{pre}}\right)}^{2}}\sqrt{\sum_{i=1}^{n}{\left(Y_{actual}-\bar{Y_{actual}}\right)}^{2}}}$$

#### 4.3.3. Model Training Process

In this study, a single-output training strategy was employed, in which an independent ANN model was trained separately for each output parameter. This approach allows for the establishment of a one-to-one mapping between each input and its corresponding output, thereby improving both the predictive accuracy and generalizability of the model. The performance metrics for each predictive model are summarized in Table 8.

To further evaluate the generalization capability of the model for different fiber types, and to examine the impact of imbalanced data distribution (e.g., the number of PVA data points being significantly larger than those of PE and PP) on the prediction errors, stratified error analyses were performed separately for the PVA and PE datasets. Independent models were trained using the PVA and PE data subsets, respectively, and four error metrics (RMSE, R^2^, R, and MAE) were calculated for both the training and test sets, as summarized in Table 9 and Table 10. The results indicate that, although the amount of PE data is relatively limited, the corresponding model still exhibits good prediction accuracy in both training and testing, confirming its applicability and stability for the PE fiber system. In addition, since the PP fiber category contains only one data point and thus lacks representative samples, it was not included in the model training or in the scope of conclusion generalization in this study.

**Figure 22:**
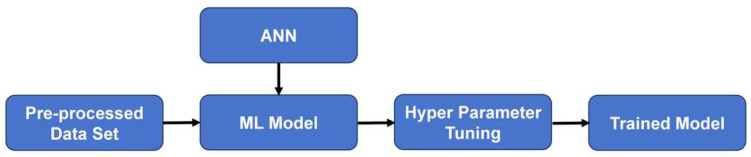
Training procedure of the model.

The dataset was divided into a training set and a testing set. The training set was primarily used for model learning, while the testing set was employed to independently evaluate the model’s generalization capability. In this study, multiple rounds of random splitting and repeated training were performed to ensure the robustness of the results. Ultimately, 70% of the data was assigned to the training set and 30% to the testing set. The training process is illustrated in Figure 22.

### 4.4. Prediction Results and Sensitivity Analysis of the ANN Model

#### 4.4.1. Prediction Results of the Model

**Figure 23:**
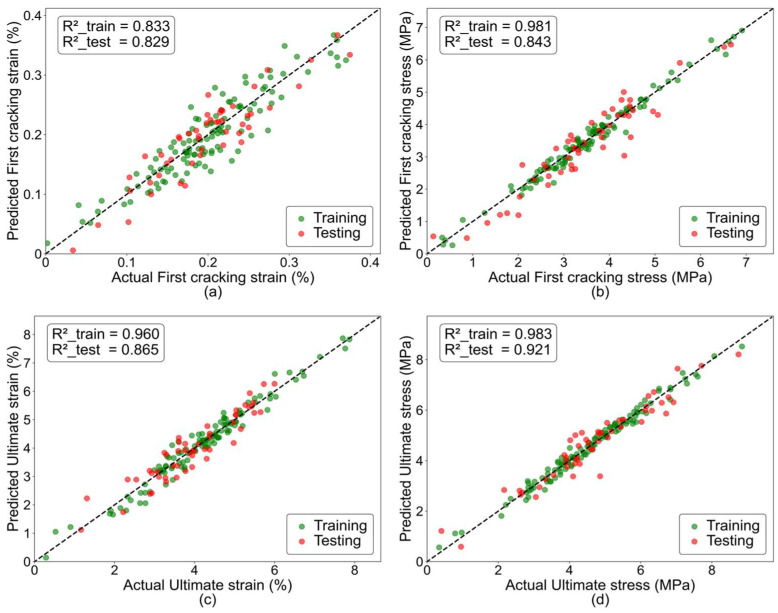
Comparison between experimental and predicted values.

The ANN prediction model developed in this study uses 10 input parameters, which encompass both the composition of the ECCs matrix and the characteristics of the fibers, to predict four essential parameters of the bilinear tensile stress–strain curve: first cracking strain, first cracking stress, ultimate strain, and ultimate stress. The model’s accuracy was evaluated by conducting separate predictions on the training and testing datasets. Evaluation metrics included R^2^, R, RMSE, and MAE. The R^2^ value reflects the model’s ability to explain the variance present in the data, where values closer to 1 indicate higher predictive accuracy. R measures the linear relationship between the predicted and actual values, with values closer to 1 denoting a better fit. Lower RMSE and MAE values indicate improved predictive precision. A comparison between the predicted and actual values for both the training and testing sets was conducted, and the corresponding curves are presented in Figure 23.

The results show that the RMSE values of first cracking strain, first cracking stress, ultimate strain, and ultimate stress for the training set are 0.147487, 0.07286, 0.111199, and 0.068835, respectively. For the testing set, the corresponding RMSE values are 0.145616, 0.086989, 0.125152, and 0.086209. These values are all relatively low, indicating that the prediction errors remain small. Moreover, the RMSE values for the training and testing sets are very close, indicating that the predictive model did not exhibit any noticeable over-fitting.

In terms of R^2^, all values for the training set exceed 0.83, with the R^2^ for ultimate strain reaching 0.982996, indicating that the model provides a high level of accuracy in predicting ultimate strain. For the testing set, all R^2^ values are above 0.82, slightly lower than those of the training set. As for R, all output parameters in both the training and testing sets exceed 0.91, demonstrating a strong linear correlation between predicted and measured results. Additionally, the MAE remains low across both datasets, indicating that the error levels are within a reasonable range. Overall, the ANN model demonstrates good accuracy in predicting key tensile parameters of ECCs and is suitable for modeling its tensile constitutive behavior.

#### 4.4.2. Sensitivity Analysis

A sensitivity analysis [112], was conducted to investigate the influence of different components on the tensile performance of ECCs. The analysis focused on four key tensile parameters: first cracking strain, first cracking stress, ultimate strain, and ultimate stress. In this method, each input parameter, such as cement, fly ash, sand, and water, was sequentially removed. The model was then retrained, and the RMSE for each output parameter was recalculated. The resulting changes in RMSE were subsequently used to evaluate the importance of each parameter in influencing the prediction outcomes. If the RMSE of the model increased significantly, it indicated that the corresponding parameter had a strong influence on prediction performance. Based on the results of all evaluated indicators, the input parameters were ranked in order of their sensitivity, allowing the identification of the component that had the greatest impact on the tensile properties of ECCs. The final results of the sensitivity analysis for first cracking strain, first cracking stress, ultimate strain, and ultimate stress are presented in Table 11.

Based on the analysis results, fiber strength and fiber content were identified as the two most critical parameters influencing the tensile performance of ECCs. Specifically, fiber strength had the greatest effect on first cracking strain and ultimate strain, whereas fiber content showed the strongest influence on first cracking stress and ultimate stress. This study offers a valuable reference for optimizing ECCs performance. By selecting high-strength fibers and appropriately adjusting fiber content, the tensile behavior of ECCs can be significantly improved without modifying the matrix mix proportions.

### 4.5. Experimental Validation and Discussion

To evaluate the accuracy and applicability of the ANN model, three additional sets of uniaxial tensile tests were conducted as the validation group, with each set comprising six dog-bone specimens. In these validation tests, the fiber content for each type was fixed at 1%. The water-to-binder ratio, sand-to-binder ratio, fly ash-to-cement ratio, and all experimental and testing procedures were kept identical to those used in the original experiments. The results of the validation group are summarized in Table 12.

**Figure 24:**
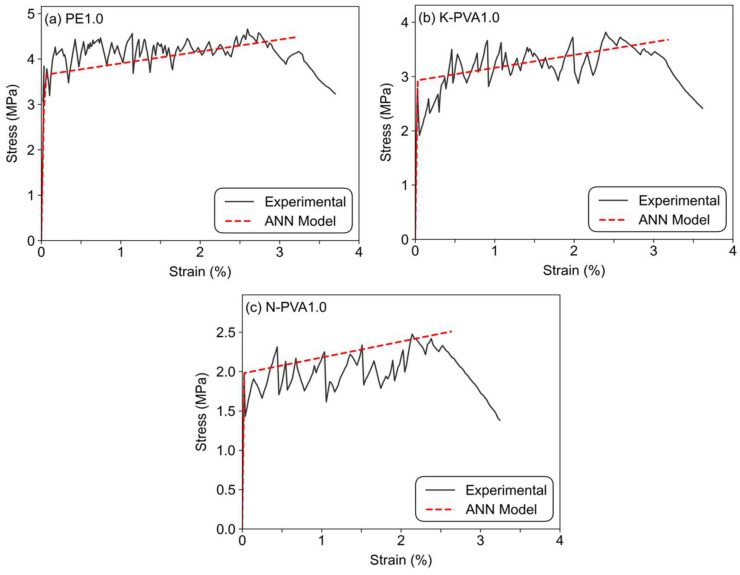
Comparison between experimental and fitted curves of the validation group.

The key parameters of the validation group, including first cracking strain, first cracking stress, ultimate strain, and ultimate stress, were calculated using the ANN model developed in this study, and the results are presented in Table 13. Figure 24 illustrates the comparison between the experimental stress–strain curves of the validation specimens and those predicted by the ANN model.

The results of the validation group demonstrate that the ANN model accurately predicts the tensile performance indicators of ECCs incorporating different fiber types. The validation results showed that the ANN model achieved great prediction performance, with relative errors between predicted and measured values for first cracking stress, ultimate strain, and ultimate stress all within 7%. This indicates that the model provides stable predictions in terms of both strength and ductility. The results also confirm the accuracy and applicability of the proposed ANN model in predicting tensile performance, offering a valuable reference for practical engineering applications.

To further improve the prediction accuracy and generalization capability of the model, future studies will focus on expanding the dataset to enhance sample diversity and training stability. In addition, ML approaches other than ANN may be introduced to explore the applicability and advantages of different algorithms in predicting ECCs performance. Moreover, integrating physical knowledge into the model development process could enhance the rationality and interpretability of the predictions. This research approach can also be applied to the performance prediction of other types of composite materials, providing theoretical support and a methodological pathway for high-performance material design and multi-parameter optimization.

## 5. Conclusions

This study conducted a systematic experimental investigation on ECCs specimens reinforced with different types of fibers: PE fibers, K-PVA fibers, and N-PVA fibers. Combined with an ANN prediction model, the study provided an in-depth analysis of the uniaxial tensile behavior of ECCs. Through macro-scale mechanical testing and micro-scale fracture surface observation, the effects of different fiber systems on the mechanical properties and failure mechanisms of ECCs were revealed. Meanwhile, based on both literature data and experimental results, a high-precision ANN model was established to predict the tensile constitutive behavior of ECCs. Sensitivity analysis was also performed to identify the key parameters influencing ECCs performance. The main conclusions are as follows:

(1) The compressive and flexural properties of ECCs were significantly affected by fiber type and fiber content. In specimens reinforced with PE fibers, compressive strength increased from 49.70 MPa at 1.3% fiber content to 54.30 MPa at 1.7%, while flexural strength improved from 10.76 MPa to 12.85 MPa. Both compressive and flexural strengths exhibited notable improvements, which can be attributed to the high tensile strength, high elastic modulus, and excellent dispersion of the PE fibers. For ECCs incorporating K-PVA fibers, the compressive strength at 1.5% content was 4.31% higher than that of 1.3%, but a further increase to 1.7% resulted in a reduction of approximately 5.35%. Meanwhile, the flexural strength exhibited a consistent upward trend, increasing from 5.63% to 8.78% as the fiber content rose. In contrast, ECCs with N-PVA fibers showed improved compressive strength at 1.3% and 1.5% content, but a marked decline at 1.7%, decreasing from 51.65 MPa to 45.57 MPa. The flexural strength showed only a slight increase, from 8.67 MPa to 9.17 MPa. Overall, PE fibers demonstrated the most pronounced enhancement in both compressive and flexural properties of ECCs, whereas the reinforcing effects of PVA fibers were relatively weaker, particularly at higher content, where excessive fiber addition led to performance degradation.
(2) Tensile test results showed that both fiber type and content have a significant influence on the tensile performance of ECCs. As the PE fiber content increased from 1.3% to 1.7%, the first cracking strength and tensile strength increased by approximately 7.23% and 11.46%, respectively, demonstrating the most effective reinforcing performance. At a content of 1.7%, K-PVA fibers increased the first cracking strength and tensile strength by about 33.44% and 19.57%, respectively, indicating a favorable bridging effect. In contrast, at higher content, N-PVA fibers led to reductions in both first cracking strength and tensile strength due to poor fiber dispersion. This suggests that the reinforcing effect is constrained by fiber distribution and interfacial bonding capacity. Therefore, PE fibers demonstrated the most effective enhancement in tensile behavior, followed by K-PVA fibers, while N-PVA fibers showed relatively weaker reinforcement effects.
(3) Fractographic analysis of fiber fracture surfaces revealed that the fiber type significantly affected the microscopic failure mechanisms and energy dissipation modes of ECCs materials. For ECCs specimens incorporating PE fibers, fiber pull-out from the matrix was the predominant feature, indicating relatively weak interfacial bonding between the fibers and the matrix. The energy dissipation was mainly dominated by the pull-out process, which contributed to improved matrix toughness. In the case of K-PVA fibers, surface modification enhanced the interfacial bonding strength. During tensile loading, they not only interacted effectively with the matrix and suppressed crack propagation, but also showed pronounced plastic deformation and splitting features. These features indicate a synergistic energy dissipation mechanism involving the coexistence of fiber pull-out and rupture. By contrast, N-PVA fibers showed poor dispersion and weak interfacial bonding, which limited their bridging capability at crack locations. As a result, the dominant energy dissipation mode was early-stage brittle fracture, offering limited effectiveness in crack suppression. In summary, the interfacial bonding strength between fibers and the matrix, the uniformity of fiber dispersion, and the deformation and failure characteristics of fibers under tensile loading are critical factors that govern the fracture mechanisms and toughness enhancement of ECCs materials.
(4) The ANN prediction model developed based on both literature and experimental data was able to accurately predict the bilinear tensile constitutive behavior of ECCs. By inputting ten material-related parameters, the model effectively predicted first cracking strain, first cracking stress, ultimate strain, and ultimate stress, demonstrating a high level of prediction accuracy. Sensitivity analysis revealed that fiber content and fiber strength were the most influential factors affecting ECCs tensile performance, while other matrix components had relatively limited impact. In addition, the ANN model was experimentally validated and successfully predicted key parameters for the validation group. The results indicate that the ANN model can accurately predict the tensile behavior of ECCs materials, thereby providing a reference basis for material design and multi-parameter optimization. However, the model still presents certain limitations, including insufficient dataset size and the lack of incorporation of physical information. Future studies should aim to expand the dataset, explore a broader range of ML algorithms for comparative modeling, and integrate physical knowledge into the modeling process to further improve the accuracy and applicability of the predictions.

## Figures and Tables

**Table 1:** Chemical compositions of cement and fly ash (wt.%).

Constituents	Cement	Fly Ash
SiO_2_	15.41	58.25
Al_2_O_3_	4.43	26.45
Fe_2_O_3_	4.91	2.50
CaO	61.54	5.60
MgO	0.72	0.93
Na_2_O	0.20	0.20
K_2_O	0.50	0.15
TiO_2_	0.60	0.05
Loss on ignition	2.88	3.2

**Table 2:** Physical and mechanical properties of fibers.

Fiber Type	Diameter (μm)	Length (mm)	Tensile Strength (MPa)	Density (g/cm^3^)	Elastic Modulus (GPa)
PE	21.6	12	3120	0.97	126
K-PVA	31	12	1600	1.3	41
N-PVA	31	12	1450	1.3	37.5

**Table 3:** Mix proportions of ECCs.

Mix ID	Cement (kg/m^3^)	Fly Ash (kg/m^3^)	Water (kg/m^3^)	River Sand (kg/m^3^)	Superplasticizer (kg/m^3^)	Fiber Type	Fiber Content (vol %)
PE1.3	555.3	666.4	329.9	439.8	8.6	PE	1.3
PE1.5	555.3	666.4	329.9	439.8	8.6	PE	1.5
PE1.7	555.3	666.4	329.9	439.8	8.6	PE	1.7
K-PVA 1.3	555.3	666.4	329.9	439.8	8.6	K-PVA	1.3
K-PVA 1.5	555.3	666.4	329.9	439.8	8.6	K-PVA	1.5
K-PVA 1.7	555.3	666.4	329.9	439.8	8.6	K-PVA	1.7
N-PVA 1.3	555.3	666.4	329.9	439.8	8.6	N-PVA	1.3
N-PVA 1.5	555.3	666.4	329.9	439.8	8.6	N-PVA	1.5
N-PVA 1.7	555.3	666.4	329.9	439.8	8.6	N-PVA	1.7

**Table 4:** Experimental results.

Specimens Group	σ_cr_/MPa	σ_p_/MPa	ε_tu_/%
PE1.3	4.15 ± 0.24	5.06 ± 0.28	3.36 ± 0.23
PE1.5	4.3 ± 0.26	5.26 ± 0.32	3.39 ± 0.33
PE1.7	4.45 ± 0.31	5.64 ± 0.36	3.55 ± 0.28
K-PVA1.3	3.11 ± 0.30	4.19 ± 0.39	3.03 ± 0.32
K-PVA1.5	3.72 ± 0.42	4.73 ± 0.38	3.19 ± 0.21
K-PVA1.7	4.15 ± 0.31	5.01 ± 0.35	3.33 ± 0.32
N-PVA1.3	2.11 ± 0.31	2.61 ± 0.25	2.56 ± 0.14
N-PVA1.5	3.13 ± 0.37	3.57 ± 0.34	3.11 ± 0.50
N-PVA1.7	2.21 ± 0.33	2.96 ± 0.23	2.96 ± 0.24

**Table 5:** Statistical details of input parameters.

Input Parameters	Unit	Range	Mean	Median	Mode	Standard Deviation	Sample Variance	Kurtosis	Skewness
Cement	kg/m^3^	190–1218	704	725.08	641.64	171.33	29,355.11	6.2	5.03
Fly ash	kg/m^3^	109–1644.86	876.93	984.07	919.86	255.98	65,524.05	1.22	3.73
Sand	kg/m^3^	129–1237.67	683.34	733.45	713.72	184.78	34,143.03	14.02	3.18
Water	kg/m^3^	185–726.73	455.87	389.09	370.55	90.29	8151.98	−1.15	0.69
Superplasticizer	kg/m^3^	0–156.18	78.09	85.23	101.62	26.03	677.56	14.05	1.96
Fiber Content	kg/m^3^	6.41–48	27.2	26.76	30.8	6.93	48.05	6.71	0.68
Length of Fiber	mm	8–13.0	10.5	9.69	9.06	0.83	0.69	10.01	7.41
Diameter of Fiber	μm	8–200	104	98.53	97.51	32	1024	6.44	3.44
Nominal strength of Fiber	MPa	626–3000	1813	1934.1	1857.02	395.67	156,552.11	14.74	7.46
Elastic modulus of Fiber	GPa	6–210	108	114.02	111.59	34	1156	9.98	2.54

**Table 6:** Statistical distribution of different fiber types in the dataset.

Sr. No.	Types of Fibers	No of Data Points	Reference and Experimental Test Data
1	Polyvinyl alcohol Fiber (PVA)	125	[67,68,69,70,71,72,73,74,75,76,77,78,79,80,81,82,83,84,85,86,87,88,89,90,91,92,93,94,95,96,97,98]
2	Polyethylene Fiber (PE)	42	[4,37,99,100,101,102,103,104,105,106]
3	Polypropylene Fiber (PP)	1	[107]

**Table 7:** Optimal hyperparameters of the ANN model.

Hyperparameters	Range	The Optimal Value for Different Parameters			
		First Cracking Strain	First Cracking Stress	Ultimate Strain	Ultimate Stress
Hidden layer size	1–100	11	44	78	44

**Table 8:** Performance indicators of the prediction model.

Set	Evaluation	First Cracking Strain	First Cracking Stress	Ultimate Strain	Ultimate Stress
Training	RMSE	0.147487	0.07286	0.111199	0.068835
	R^2^	0.833297	0.980921	0.982996	0.960416
	R	0.952742	0.991812	0.99483	0.995462
	MAE	0.105951	0.053928	0.082305	0.050887
Testing	RMSE	0.145616	0.086989	0.125152	0.086209
	R^2^	0.829304	0.843067	0.921049	0.86507
	R	0.914375	0.947678	0.992706	0.955019
	MAE	0.107275	0.061997	0.088099	0.05698

**Table 9:** Predictive performance on the PVA dataset under different evaluation metrics.

Set	Evaluation	First Cracking Strain	First Cracking Stress	Ultimate Strain	Ultimate Stress
Training	RMSE	0.029541	0.303779	0.419238	0.268921
	R^2^	0.895024	0.974446	0.981169	0.958358
	R	0.946058	0.997910	0.990842	0.978966
	MAE	0.014254	0.155181	0.203787	0.149145
Testing	RMSE	0.030421	0.364319	0.361831	0.261071
	R^2^	0.869930	0.872923	0.862853	0.953189
	R	0.954067	0.940166	0.930410	0.977799
	MAE	0.013107	0.153993	0.165250	0.140601

**Table 10:** Predictive performance on the PE dataset under different evaluation metrics.

Set	Evaluation	First Cracking Strain	First Cracking Stress	Ultimate Strain	Ultimate Stress
Training	RMSE	0.018795	0.122708	0.318833	0.234030
	R^2^	0.862550	0.955804	0.966598	0.944258
	R	0.928736	0.977652	0.983157	0.971729
	MAE	0.007827	0.057668	0.176567	0.127093
Testing	RMSE	0.028040	0.190068	0.379368	0.270602
	R^2^	0.736408	0.863838	0.959584	0.892715
	R	0.827365	0.936994	0.998009	0.964249
	MAE	0.013500	0.122637	0.263594	0.200312

**Table 11:** Results of sensitivity analysis for each indicator.

Removed Parameter	First Cracking Strain		First Cracking Stress		Ultimate Strain		Ultimate Stress	
	RMSE	Rank	RMSE	Rank	RMSE	Rank	RMSE	Rank
Cement	0.406527	4	0.56342655	5	1.5544125	3	2.3899116	2
Fly ash	0.3105703	7	0.4924256	8	0.822701	10	1.3638533	7
Sand	0.3031504	8	0.29378688	10	1.13849062	8	0.620447	10
Water	0.34189722	5	0.5503779	7	1.12819292	9	0.963163	9
Superplasticizer	0.24652216	9	0.5555797	6	1.14126928	7	1.8987614	4
Fiber Content	0.65544883	2	1.202515	1	1.479178	4	2.65946804	1
Length of Fiber	0.32420578	6	0.7622586	3	1.7101413	2	1.3609549	8
Diameter of Fiber	0.2130584	10	0.37593833	9	1.2635776	6	1.8278564	5
Nominal strength of Fiber	0.769809	1	1.1155825	2	1.8959366	1	1.5406901	6
Elastic modulus of Fiber	0.58451957	3	0.663625	4	1.39173594	5	2.2892183	3

**Table 12:** Experimental results of the validation group.

Specimens	First Cracking Strain/%	First Cracking Stress/MPa	Ultimate Strain/%	Ultimate Stress/MPa
PE1.0%	0.036	3.84	3.24	4.66
K-PVA1.0%	0.028	2.78	3.02	3.82
N-PVA1.0%	0.023	1.98	2.53	2.36

**Table 13:** Predicted results of the validation group.

Specimens	First cracking Strain/%	First Cracking Stress/MPa	Ultimate Strain/%	Ultimate Stress/MPa
PE1.0%	0.041	3.65	3.21	4.48
K-PVA1.0%	0.029	2.93	3.19	3.68
N-PVA1.0%	0.025	1.98	2.64	2.51

## Data Availability

The original contributions presented in this study are included in the article. Further inquiries can be directed to the corresponding author.
